# A new insight to explore the regulation between *S*‐nitrosylation and *N*‐glycosylation

**DOI:** 10.1002/pld3.110

**Published:** 2019-03-01

**Authors:** Hu Du, Lichao Chen, Ni Zhan, Jinye Mu, Bo Ren, Jianru Zuo

**Affiliations:** ^1^ Vegetable Research Institute Guangdong Academy of Agricultural Sciences Guangdong Key Laboratory for New Technology Research of Vegetables Guangzhou China; ^2^ State Key Laboratory of Plant Genomics and National Plant Gene Research Center (Beijing) Institute of Genetics and Developmental Biology Chinese Academy of Sciences Beijing China; ^3^ University of Chinese Academy of Sciences Beijing China

**Keywords:** *Arabidopsis*, *DGL1*, *gsnor1‐3*, *N*‐glycosylation, *S*‐nitrosylation, *TGG2*

## Abstract

Nitric oxide (NO) is a signal molecule in plants and animals. *Arabidopsis* GSNO reductase1 (AtGSNOR1) catalyzes metabolism of *S*‐nitrosoglutathione (GSNO) which is a major biologically active NO species. The *GSNOR1* loss‐of‐function mutant *gsnor1‐3* overaccumulates GSNO with inherent high *S*‐nitrosylation level and resistance to the oxidative stress inducer paraquat (1,1′‐dimethyl‐4,4′‐bipyridinium dichloride). Here, we report the characterization of *dgl1‐3* as a genetic suppressor of *gsnor1‐3*. *DGL1* encodes a subunit of the oligosaccharyltransferse (OST) complex which catalyzes the formation of *N*‐glycosidic bonds in *N*‐glycosylation. The fact that *dgl1‐3* repressed the paraquat resistance of *gsnor1‐3* meanwhile *gsnor1‐3* rescued the embryo‐lethal and post‐embryonic development defect of *dgl1‐3* reminded us the possibility that *S*‐nitrosylation and *N*‐glycosylation crosstalk with each other through co‐substrates. By enriching glycoproteins in *gsnor1‐3* and mass spectrometry analysis, TGG2 (thioglucoside glucohydrolase2) was identified as one of co‐substrates with high degradation rate and elevated *N*‐glycosylation level in *gsnor1‐3 ost3/6*. The *S*‐nitrosylation and *N*‐glycosylation profiles were also modified in *dgl1‐3* and *gsnor1‐3*. Thereby, we propose a linkage between *S*‐nitrosylation and *N*‐glycosylation through co‐substrates.

## 1 INTRODUCTION

*S*‐nitrosylation is a crucial mechanism for the exertion of nitric oxide (NO) biological functions in animal and plants. During *S*‐nitrosylation modification, NO molecules are covalently added to consensus cysteine residues flanked by acidic and basic amino acid residues of peptides forming endogenous *S*‐nitrosothiols and modifying protein structures and functions (Greco et al., 2006; Seth & Stamler, 2011; Stamler, Toone, Lipton, & Sucher, 1997). In animals, three major isoforms of NO synthases (NOSs: iNOS, eNOS, nNOS) have been identified since 1989 (Alderton, Cooper, & Knowles, 2001) however not any NOS similar to that in animal models has been identified in plants, although the *S*‐nitrosoglutathione reductase (GSNOR) has been characterized. GSNOR irreversibly catalyzes the metabolism of GSNO which is a major biological active form of NO to GSSG and NH_3_ as main products thus indirectly controls the *S*‐nitrosylation level (Malik, Hussain, Yun, Spoel, & Loake, 2011). In *Arabidopsis*, mutations in the singled‐copied *GSNOR1* gene cause defects in development (Feechan et al., 2005; Kwon et al., 2012). The *gsnor1‐3* is a loss‐of‐function mutant in *GSNOR1* gene with increased SNO level, accompanied by severe developmental defects such as semi‐dwarf, bushy, and reduced fertility (Feechan et al., 2005; Kwon et al., 2012; Lee, Wie, Fernandez, Feelisch, & Vierling, 2008). In our previous study (Chen et al., 2009), we performed a genetic screen for the paraquat resistant mutant and characterized the *par2‐1* mutant which was identical to *gsnor1‐3/hot5*. Recent researches have shown *gsnor1‐3* plays critical roles in studying NO function and GSNOR1 regulated *S*‐nitrosylation (Feng et al., 2013; Hu et al., 2015, 2017; Tada et al., 2008; Yang et al., 2015; Zhan et al., 2018). In order to further analyze the role of *GSNOR1* in *Arabidopsis* development, we performed another genetic screen for the suppressor of *gsnor1‐3* based on its paraquat resistance. Map‐based clone revealed that the suppressor was a mutation in one subunit of the oligosaccharyltransferse (OST) complex which catalyzes the transfer of oligosaccharide onto a nascent protein in *N*‐glycosylation.

*N*‐glycosylation is a ubiquitous protein modification in eukaryotes, almost 70% of eukaryotic proteins are glycosylated (Mononen & Karjalainen, 1984). In secretory pathway, glycosylation is essential for proteins to be secreted or integrated in membranes. Nascent polypeptides in the endoplasmic reticulum (ER) lumen which are covalently attached with oligosaccharide to asparagine (Asn) side‐chains will be folded into the native structures and delivered to the Golgi apparatus for further folding and conformational maturation, while underglycosylation will cause proteins misfolding and trigger ER stress, unfolded proteins response (UPR), and ER‐associated degradation (ERAD) (Aebi, 2013; Lannoo & Van Damme, 2015; Moremen, Tiemeyer, & Nairn, 2012). The ERAD system translocates misfolded proteins across the ER membrane into the cytosol where ubiquitin‐conjugated enzymes target these misfolded proteins for degradation.

OST is a multi‐subunit complex which catalyzes oligosaccharides attached to conserved asparagine residues in Asn‐X‐Ser/Thr (N‐X‐S/T) motif of polypeptides in *N*‐glycosylation. At least seven subunits of OST have been reported in *Arabidopsis* such as STT3a, STT3b, OST3/6, DGL1, DAD1, DAD2, and HAP6 (Aebi, 2013; Farid et al., 2013; Mohorko, Glockshuber, & Aebi, 2011). These subunits are believed to exert distinct functions, for example DAD1 and DAD2 function in OST anchoring, STT3a and STT3b form the catalytic center of OST (Burda & Aebi, 1999; Gallois et al., 1997; Kelleher, Karaoglu, Mandon, & Gilmore, 2003; Mohorko et al., 2011; Nilsson et al., 2003), and DGL1 (defective glycosylation1) was supposed to bind lipid‐linked oligosaccharide donor substrates (Pathak, Hendrickson, & Imperiali, 1995). Most mutations in these subunits cause decreased glycosylation level and severe plant development defects. *STT3a* or *STT3b* knockout mutants are viable while *stt3a* is salt‐sensitive and *stt3b* has no obvious phenotype but *stt3a stt3b* double mutant is gametophytic lethal (Koiwa et al., 2003). *OST3/6* deficiency results in overall underglycosylation and hypersensitivity to salt/osmotic stress (Farid et al., 2013). The *dgl1‐1* and *dgl1‐2* mutants were isolated in a screen for mutants that harbor cell wall defects and short dark‐grown hypocotyl phenotype. Furthermore, *dgl1‐1* and *dgl1‐2* mutants were underglycosylated and *dgl1‐2* was embryo lethal (Lerouxel et al., 2005).

Here, we isolated a suppressor of *gsnor1‐3* from an ethylmethane sulfonate (EMS)‐mutagenized library based on the hypersensitive phenotype to paraquat. Positional cloning showed that the suppressor mutant nominated as *dgl1‐3* was due to a point mutation in the exon of *DGL1* which encodes a subunit of the OST complex. We hybridized *gsnor1‐3* with *dgl1‐3* and other *ost* mutants such as *stt3b* and *ost3/6*. The double mutants exhibited some intermediate phenotypes, for example *gsnor1‐3 dgl1‐3* and *gsnor1‐3 ost3/6* were sensitive to paraquat, whereas *gsnor1‐3 ost3/6* was also resistant to glycosylation inhibitor tunicamycin; the *dgl1‐3* mutant was partial embryo‐lethal and post‐embryonic development defective while the *gsor1‐3* ^*−/−*^ *dgl1‐3* ^*−/+*^ self‐pollinated F2 population contained 1/4 of *gsnor1‐3* ^*−/−*^ *dgl1‐3* ^*−/−*^ with fertility. Immunoblot analysis showed that the profiles of *S*‐nitrosylation and *N*‐glycosylation were altered in d*gl1‐3* and *gsnor1‐3* compared to that in WT, respectively. By enriching glycoproteins in *gsnor1‐3* and using mass spectrometry analysis, TGG2 (thioglucoside glucohydrolase2) was identified as one of the 26 co‐substrates of *S*‐nitrosylation and *N*‐glycosylation which was degraded faster with elevated *N*‐glycosylation level in *gsnor1‐3 ost3/6*. On the basis of our research and reported information, we speculated that disulfide may play a role as a link between *S*‐nitrosylation and *N*‐glycosylation.

## 2 MATERIALS AND METHODS

### 2.1 Plant materials, growth conditions, and genetic screen for *gsnor1‐3 dgl1‐3*

Plants were grown under a 16 hr light/8 hr dark cycle or continuous white light (120~130 μmol m^−2^ s^−1^) at 22°C in soil or on a 1/2 MS medium containing 3% sucrose and 0.8% agar.

To screen *gsnor1‐3 dgl1‐3* mutant, EMS‐mutagenized M2 seeds based on *gsnor1‐3* in the Col‐0 background were germinated and grown on 1/2 MS agar plates for 7 days then transferred to 1/2 MS with or without 0.1 μM paraquat for additional 5 days. In the first 7 days, the seedlings were grown on the vertically placed plates for elongation of roots, after transferring the seedlings were placed in the inverted orientation for bending roots. By comparing the relative bending roots lengths with and without paraquat treatment, paraquat hypersensitive mutant was identified. Genetic analyses were performed by pair‐wise crossing of individual mutants followed by assessing segregation patterns in F1 and F2 generations.

### 2.2 Genetic mapping of *dgl1‐3*

F2 seeds derived from crosses between *gsnor1‐3* ^*−/−*^ *dgl1‐3* ^*−/+*^ (Col‐0) and *gsnor1‐3* ^*−/−*^ (Ler) were germinated on 1/2 MS medium for 7 days and seedlings with dark cotyledons were selected for genetic mapping. An F2 population of about 300 mutant seedlings was analyzed to define the *dgl1‐3* mutation between K1F13‐2 and MSN2 (see Supporting information for sequences of primers). The sequences of dCAPS marker used to identify homogenous and heterozygous *dgl1‐3* were as follow (5′ to 3′): F: TATAGTCATCTACTCAATGCAAGAA; R: TTATGATTTACAGGCTCCCGTGCGCTTC.

### 2.3 Genetic complementation

A *DGL1* (At5g66680) genomic DNA fragment of 4226‐bp containing 3′‐URT was obtained by PCR from wild‐type (Col‐0) plants, verified by sequencing and then cloned into the *XhoI* and *SpeI* sites of a binary vector pER8 to yield pER8‐*DGL1*. The *Flag* or *GFP* sequence was linked to the C or N terminal of *DGL1* in a head‐to‐tail configuration. The constructed vector was transformed into both *dgl1‐3* ^*−/+*^ and *gsnor1‐3* ^*−/−*^ *dgl1‐3* ^*−/+*^ plants by floral dipping and the plants with the homogenous *dgl1‐3* mutation background in T3 generation were isolated by sequencing the PCR products amplified by primers anchored between the mutated site and 5′‐UTR.

### 2.4 Analysis of trypan blue and DAB staining

For trypan blue staining, the seedlings were stained with lactophenol‐trypan blue (10 ml of lactic acid, 10 ml of glycerol, 10 g of phenol, 10 mg of trypan blue, dissolved in 10 ml of distilled water) (Keogh, Deverall, & McLeod, 1980). The whole seedlings were boiled for approximately 1 min in the stain solution and then decolorized in chloral hydrate (2.5 g of chloral hydrate dissolved in 1 ml of distilled water) for at least 30 min. The seedlings were viewed and photographed under a stereoscope.

DAB(Beyotime, Cat#: ST003) was dissolved into a 50 mM Tris‐HCl, pH 3.8 solution with a concentration of 1 mg/ml. The seedlings were submerged into the DAB solution in dark for 5–6 hr then dehydrated in 95% ethanol (Yokawa, Kagenishi, Kawano, Mancuso, & Baluška, 2011).

#### 2.4.1 Glycoprotein enrichment

Plant material was homogenized in liquid nitrogen and extracted in extraction buffer (20 mM Tris‐HCl, pH 7.0 and 20 mM β‐mercaptoethanol). Homogenate was filtered through Miracloth and centrifuged at 10,000 *g* for 10 min. Proteins in cleared extracts were precipitated by 80% saturation of ammonium sulfate and centrifugation at 10,000 *g* for 10 min at 4°C. Protein precipitates were resuspended in column buffer (20 mM Tris‐HCl, pH 7.0, and 500 mM NaCl), centrifuged, and loaded onto Con A‐Sepharose (Nanocs, Cat#: AR‐CAN‐1, 1‐ml bead volume) equilibrated with column buffer. After washing with column buffer, bound proteins were eluted with 1% SDS and analyzed by 15% SDS‐PAGE and detected by Coomassie Brilliant Blue staining (CBS) (Koiwa et al., 2003).

#### 2.4.2 Mass spectrometry analysis

The enriched glycoproteins in gels were digested by trypsin (0.01 mg/ml; Promega, Cat #: V5111) (Hu et al., 2015). The digested peptides were sent to National Center for Protein Sciences (Beijing) (http://www.phoenix-center.cn) for analyzing by mass spectrometer (Thermo Q Exactive, USA).

Raw data were used for a search against *Arabidopsis* protein database (www.ncbi.nlm.nih.gov/guide/proteins/; Version, Aug 8, 2018) with the taxonomy restriction to “*Arabidopsis thaliana*”. The BioWorks TurboSequest software was used for the database searching using the following parameters: the mass tolerances for peptides and fragment ions were set to 0.5 Da; (FDR) < 0.05; PSM FDR, Protein FDR, and Site FDR were set under 0.01; minima peptide length was 6; top MS/MS peaks per 100 Da (TOF) was 10.

#### 2.4.3 Biotin‐switch and Western blot assay

*S*‐nitrosylated proteins were analyzed by the biotin‐switch assay as described previously (Feng et al., 2013; Yang et al., 2015).

For Western blot analysis, 7‐day‐old seedlings were homogenized in a denaturating buffer [20 mM Tris‐HCl, pH 6.8, 0.3% β‐mercaptothanol, 5% (v/v) glycerol, and 1% (w/v) SDS]. After boiling for 5 min, the proteins were separated by SDS‐PAGE in 8% or 15% polyacrylamide gels. Polypeptides were then transferred onto a PVDF membrane. For immunodetection, PVDF membranes were probed with HRP antibody (GenScript, Cat#: A00619) raised against β(1,2)‐xylose and α(1,3)‐fucose *N*‐glycan residues (Lerouxel et al., 2005).

## 3 RESULTS

### 3.1 Genetic screen for the suppressor of *gsnor1‐3*

**Figure 1:**
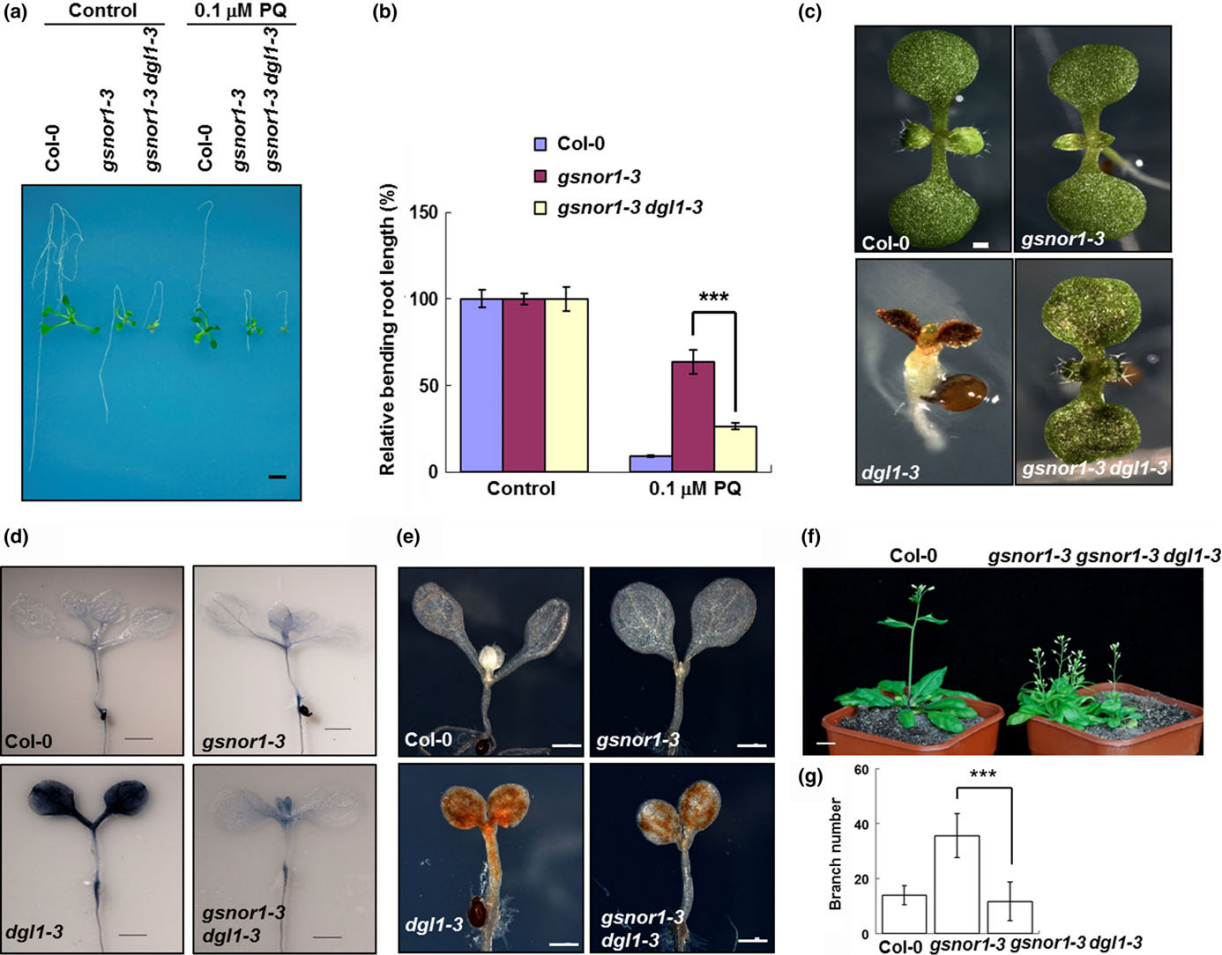
Genetic screen and phenotype analysis of *gsnor1‐3 dgl1‐3* and *dgl1‐3*. (a) Bending root growth of 7‐day‐old seedlings cultured for additional 5 days on 1/2 MS (Control) and 1/2 MS with 0.1 μM paraquat (0.1 μM PQ). All seedlings and plants exhibited in this figure and below were homogenous background. Bar = 5 mm. (b) Relative bending root length of 7‐day‐old seedlings cultured for additional 5 days on 1/2 MS (Control) and 0.1 μM paraquat (0.1 μM PQ). Asterisks indicate significant differences (***p < 0.001, Student's *t* test). (c) Cotyledons of 7‐day‐old seedlings. Bar = 0.1 mm. (d) Typan blue staining of cotyledons of 7‐day‐old seedlings. Bars = 0.2 mm. (e) DAB staining for hydrogen peroxide in cotyledons of 7‐day‐old seedling. Bars = 0.2 mm. (f) 40‐day‐old Col‐0, *gsnor1‐3* and *gsnor1‐3 dgl1‐3* plants in greenhouse. The backgrounds of plants have been identified by genotyping. Bar = 1 cm. (g) Branch number of 60‐day‐old plants grown in greenhouse. Asterisks indicate significant differences (***p < 0.001, Student's *t* test)

Paraquat (PQ) is a kind of nonselective herbicide which has an efficient inducer of cell death in animal and plant cells (Suntres, 2002). In our previous research, we got a *par2‐1* mutant with strong paraquat resistance which is allelic to *gsnor1‐3/hot5* (Chen et al., 2009). The *gsnor1‐3/hot5/par2‐1* mutant showed anti‐cell death, reduced fertility and heat acclimation phenotypes. We hypothesized that mutations render *gsnor1‐3* sensitive to paraquat may represent important genetic loci that are involved in regulation of cell death or NO metabolism. Therefore, we carried out a genetic screen for paraquat sensitive mutants by surveying ethylmethane sulfonate (EMS)‐generated library based on *gsnor1‐3*. As the mutants after paraquat treatment should be feeble and require to recover, a low concentration of paraquat and delicate method were used for screening the mutants (Figure S1, Supporting Information). About 8,000 lines of seeds in the library were germinated on 1/2 MS medium vertically for 7 days then transferred to 1/2 MS mediums with or without 0.1 μM paraquat and invertly placed and cultured for additional 5 days. The bent roots lengths of seedlings grown on mediums were measured and the relative root lengths were calculated with roots grown on 1/2 MS medium without PQ as controls. After screening, we got a double mutant named *gsnor1‐3 dgl1‐3* based on the gene cloned in the mutant (Figure 1a,b). Indeed, *gsnor1‐3 dgl1‐3* was so tender that high humidity or pests in greenhouse caused plant death easily. Therefore we crossed *gsnor1‐3* ^*−/−*^ *dgl1‐3* ^*−/+*^ background plants (developed normally) selected from original M2 population with *gsnor1‐3* ^*−/−*^ and Col‐0 separately to obtain *gsnor1‐3* ^*−/−*^ *dgl1‐3* ^*−/−*^ and *dgl1‐3* ^*−/−*^ mutants in F2 populations.

### 3.2 The phenotypes of *dgl1‐3* and *gsnor1‐3 dgl1‐3* mutants

Besides the sensitivity to paraquat, the cotyledons of both *dgl1‐3* and *gsnor1‐3 dgl1‐3* were dark, suggesting accumulation of anthocyanin (Figure 1c). Trypan blue staining showed that the cell death level was much higher in *dgl1‐3* than in *gsnor1‐3 dgl1‐3* (Figure 1d). The hydrogen peroxide level of *dgl1‐3* was also depressed in *gsnor1‐3 dgl1‐3* detected by 3,3‐diaminobenzidine (DAB) staining (Figure 1e). After approximately 12 days of culture on 1/2 MS medium *dgl1‐3* was dead indicating a post‐embryonic development cease phenotype as reported by Lerouxel et al. (2005), while *gsnor1‐3 dgl1‐3* survived and bloomed, and few seeds were obtained (Figure 1f). The phenotype of *gsnor1‐3 dgl1‐3* double mutant still looks like *gsnor1‐3* with dwarf and fertility defective but fewer branches (Figure 1g).

Among the F2 population of self‐pollinated *gsnor1‐3* ^*−/−*^ *dgl1‐3* ^*−/+*^ seedlings, the dark cotyledons phenotype was segregated in a 1:3 ratio (dark: green = 34: 125, χ^2^ = 1.1), indicating that the mutation is recessive in a single nuclear gene. But the F2 of self‐pollinated *dgl1‐3* ^*−/+*^ segregated dark cotyledons seedlings less than 25% of total, fluctuated from 8% to 15%. In fact, when we used *gsnor1‐3* ^*−/−*^ *dgl1‐3* ^*−/+*^ (Col‐0 background) to cross *gsnor1‐3* ^*−/+*^ (Landsberg *erecta* background) to generate F2 population for mapping, we noticed that the segregation ratio was 1:3 in *gsnor1‐3* ^*−/−*^ background population but in other background populations the number of dark cotyledons seedlings was less than that in theory. Thus the *dgl1‐3* mutant was embryo lethal as reported (Lerouxel et al., 2005) while *gsnor1‐3* rescued the defect. The phenotypes of *dgl1‐3* ^*−/−*^ and *gsnor1‐3* ^*−/−*^ *dgl1‐3* ^*−/−*^ indicated an interactive genetic regulation between *S*‐nitrosylation and *N*‐glycosylation.

### 3.3 Molecular cloning of *DGL1*

**Figure 2:**
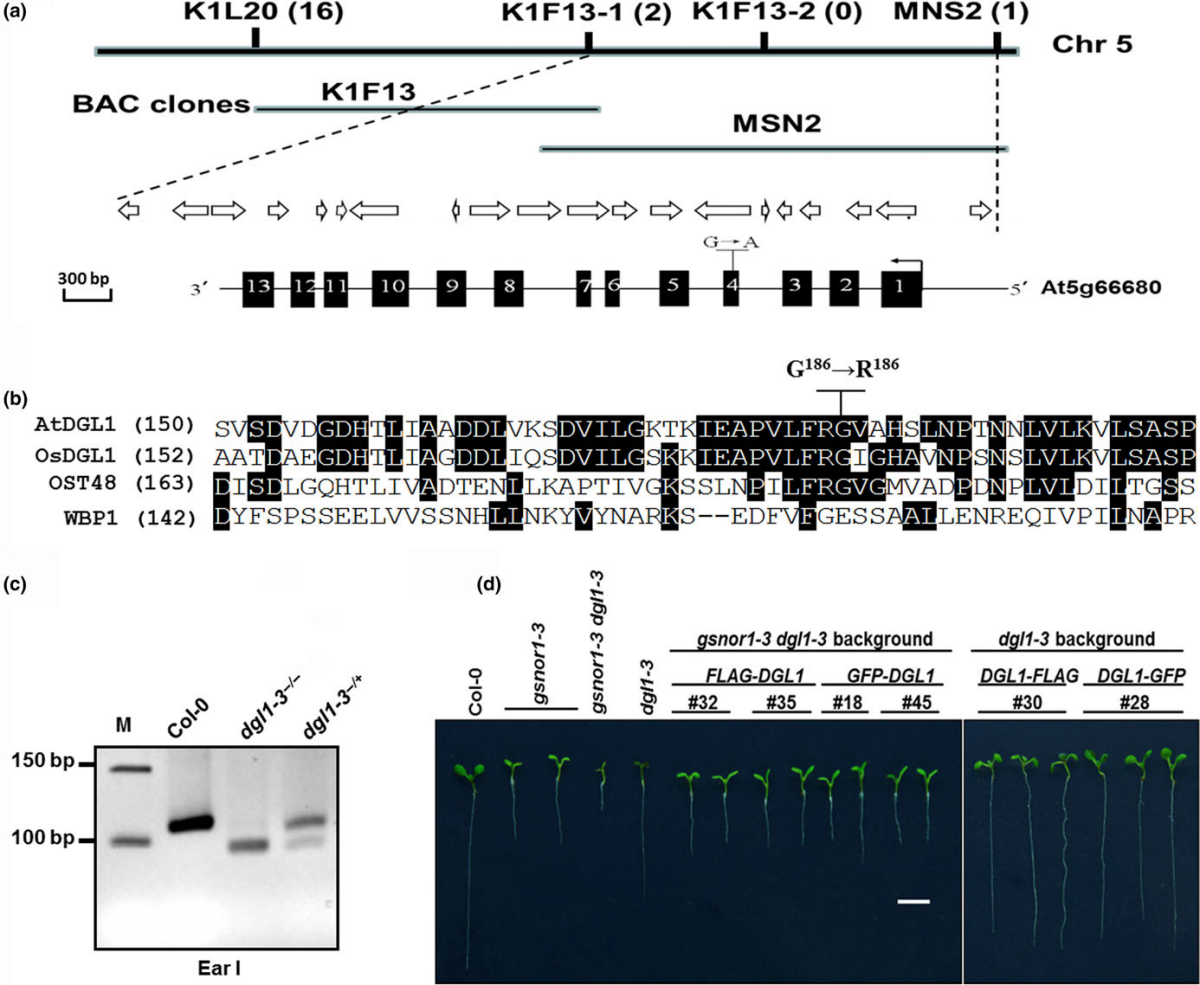
Molecular characterization of the *dgl1‐3* mutation. (a) A schematic map of positional cloning of *dgl1‐3*. Top: region with markers used in genetic mapping. Numbers in parentheses indicate recombinants identified in the mapped population of given marker. Middle: annotated genes between markers K1F13‐1 to MNS2. Arrows and spaces denote the transcribed direction and distance of each gene. Bottom: structure of ***DGL1.*** Exons and introns are represented by filled boxes and solid lines. The position of mutated nucleotide is shown. (b) Amino acid sequences of DGL1 and its homologs from different species. The position of mutated residue is shown. Conserved residues are shaded. Accession numbers: Arabidopsis (AtDGL1): NP_569038.1; Rice (OsDGL1): XP_015646882; Human (OST48): NP_005207.2; Yeast (WBP1): NP_010914.3. (c) Electrophoresis profiles of dCAPS products. The PCR products were digested by restricted enzyme EarI. M: DNA marker. Molecular size of DNA fragments were 114 bp (Col‐0) and 100 bp *(dgl1‐3* ^−/−^ *)*. (d) Molecular complementation of the *dgl1‐3* and *gsnor1‐3 dgl1‐3* mutants. Seedlings with different backgrounds were germinated and grown on 1/2 MS medium for 7 days

Using the *gsnor1‐3* ^*−/−*^ background seedlings with dark cotyledons in the F2 population obtained from hybridization of *gsnor1‐3* ^*−/−*^ *dgl1‐3* ^*−/+*^ (Columbia) × *gsnor1‐3* ^*−/+*^ (*Landsberg erecta*), we mapped the mutation on chromosomes V. By monitoring genetic recombination in a population of about 300 mutant seedlings, we located *dgl1‐3* in a ~70 kb region containing 20 open reading frames. DNA sequencing analysis of all 20 genes revealed a mutation in At5g66680, characterized as a G‐to‐A transition in exon 4, which converts a glycine (G) into an arginine acid (R) at residue 186 (Figure 2a,b). This glycine residue is highly conserved in related proteins across plants and animals (Figure 2b). According to the nucleic acid transition of G to A, we designed a dCAPS marker to distinguish homozygous and heterozygous *dgl1‐3* mutant (Figure 2c).

To verify the identity of the candidate *DGL1* gene, we performed a genetic complementation experiment. A 3 × Flag or GFP tag sequence was connected with a 4226‐bp wild‐type genomic DNA fragment which contains the putative promoter region, 5′‐untranslated region (UTR), coding sequence of At5g66680, and was cloned into a binary vector. The resulting construct was transformed into *dgl1‐3* ^*−/+*^ and *gsnor1‐3* ^*−/−*^ *dgl1‐3* ^*−/+*^ background plants by floral dipping. Among the analyzed transgenic lines in T3 generation with homogenous *dgl1‐3* mutation, all seedlings showed a phenotype similar to that of *gsnor1‐3* or Col‐0 (Figure 2d). But the Flag or GFP tag in these transgenic lines could not be detected by Western blot, neither of the tags were linked to the C or N terminal while the integration of *DGL1* into the genome was confirmed by conventional PCR analyses (Figure S2, Supporting Information). Previous researches have reported that human OST48 is allelic to DGL1 and consists of 456 residues with the first 42 residues in N terminal including a signal sequence meanwhile the nine residues in the C terminal constitutes a cytosolic segment which interacts with DAD1 (Fu, Ren, & Kreibich, 1997; Mohorko et al., 2011). Pig OST48 contains a double lysine motif at the very C terminus which was suggested to confer ER residency (Hardt, Aparicio, & Bause, 2000; Hardt, Aparicio, Breuer, & Bause, 2001; Mohorko et al., 2011). Because of the homolog in function and structure between DGL1 and OST48, we believed some special structure or functions might be possessed by both terminals of DGL1 and the tags in our transgenic lines possibly be sheared before protein maturation.

### 3.4 The *gsnor1‐3* mutation endows OST mutants with tunicamycin resistance

**Figure 3:**
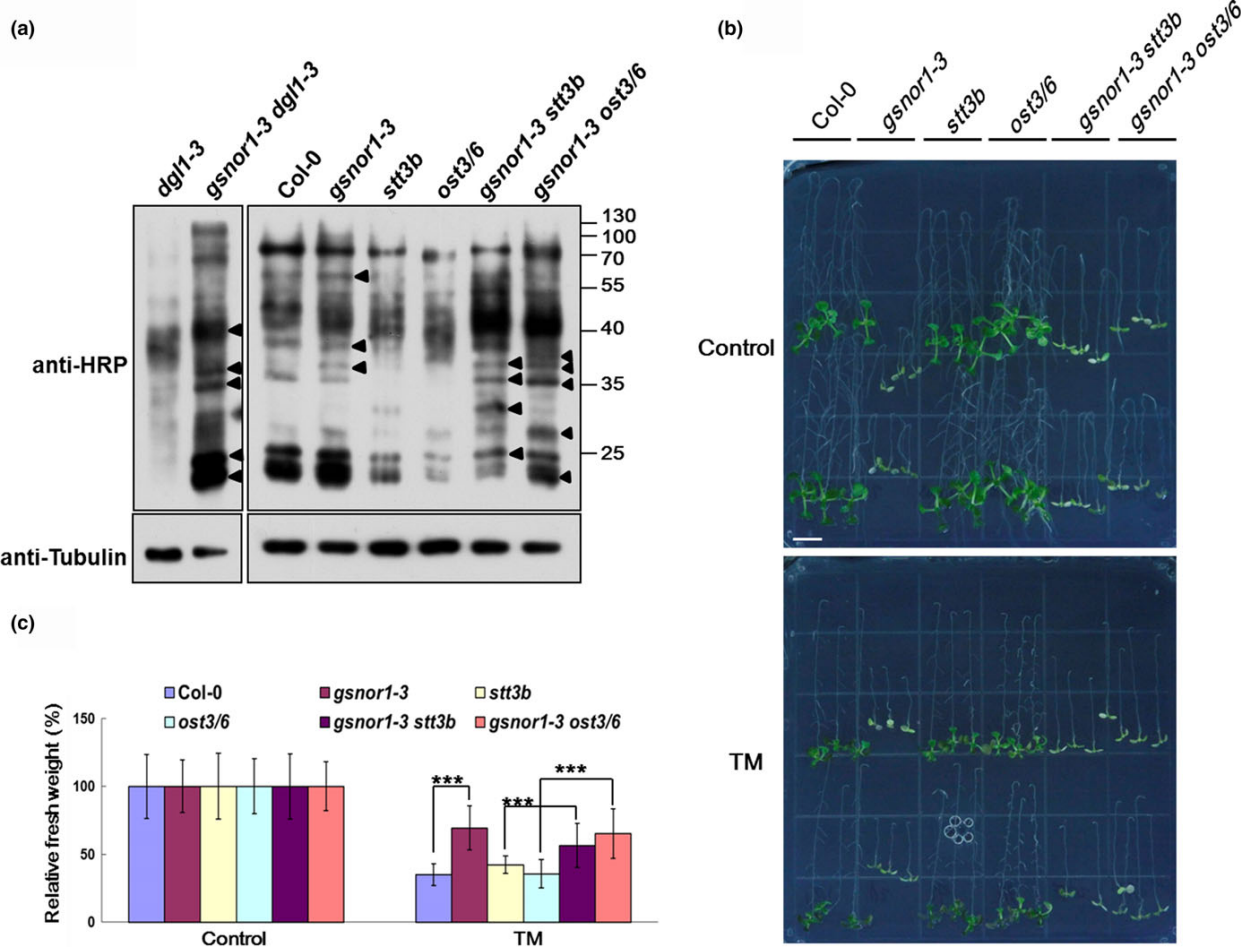
*N*‐glycosylation pattern and tunicamycin resistance analysis. (a) Immunoblotting with antibody (HRP) raised against β(1,2)‐xylose and α(1,3)‐fucose *N*‐glycan epitopes. Arrowheads show altered glycoproteins in *gsnor1‐3* and *gsnor1‐3 ost* double mutants compared to that in Col‐0 and relative *ost* mutants. (b) 7‐day‐old seedling germinated and vertically grown on 1/2 MS medium were transferred to 1/2 MS medium without (Control) or with 0.5 mg/ml tunicamycin (TM) in inverted orientation for additional 5 days. Bar = 0.5 cm. (c) Relative fresh weight of per plant in panel (b). Asterisks indicate significant differences (***p < 0.001, Student's *t* test)

*DGL1* encodes a critical subunit of the OST complex which catalyzes transfer of oligosaccharide onto nascent peptides in *N*‐glycosylation. The mutation of *DGL1* causes the underglycosylation of proteins. Immunoblot analysis using a Horseradish Peroxidase (HRP) antibody specific for α‐(1,3)‐fucose and β(1,2)‐xylose *N*‐linked glycan epitopes showed different profiles in *dgl1‐3* and *gsnor1‐3 dgl1‐3* (Figure 3a left, arrowheads). Actually, the bands which represented glycoproteins were hardly to be detected in *dgl1‐3* by HRP antibody indicating a mess of glycosylation in *dgl1‐3* which was recovered in *gsnor1‐3 dgl1‐3*.

The OST complex consists of at least eight subunits in *Arabidopsis*, we crossed *stt3b* (SALK_134449C) and *ost3/6* (SALK_067271C) with *gsnor1‐3*. Unfortunately the seeds of *dgl1* SALK lines obtained from donators were inactive or without mutation. All those related mutations and Col‐0 were also detected by immunoblot analysis with the HRP antibody. The immunoblot showed that the profiles of glycoproteins in *gsnor1‐3* were differently expressed to that in Col‐0; meanwhile the depression of glycosylation in *stt3b* and *ost3/6* were highly elevated in double mutants (Figure 3a right, arrowheads).

Tunicamycin (TM) is a Streptomyces‐derived inhibitor of eukaryotic protein *N*‐glycosylation and also an inducer of endoplasmic reticulum (ER) stress. Plants treated with TM will accumulate unglycosylated, misfolded proteins in ER. We tested if *gsnor1‐3* endowed the *stt3b* and *ost* mutants resistance to TM. An experiment similar to screening for the *gsnor1‐3* suppressor was carried out but TM inhibition was so strong that the bending roots were ceased to elongate, thus we measured the fresh weight of per plant (Figure 3b). The relative fresh weight of *gsnor1‐3* after TM treatment was higher than that of Col‐0 indicating a strong resistance to TM. Similarly all the *gsnor1‐3 ost* double mutants showed higher resistance than relative *ost* single mutants (Figure 3c). From these experiments we have reasons to believe that the elevated *S*‐nitrosylation of proteins promoted *N*‐glycosylation.

### 3.5 The S‐nitrosylaiton pattern was strongly changed in *gsnor1‐3 dgl1‐3*

**Figure 4:**
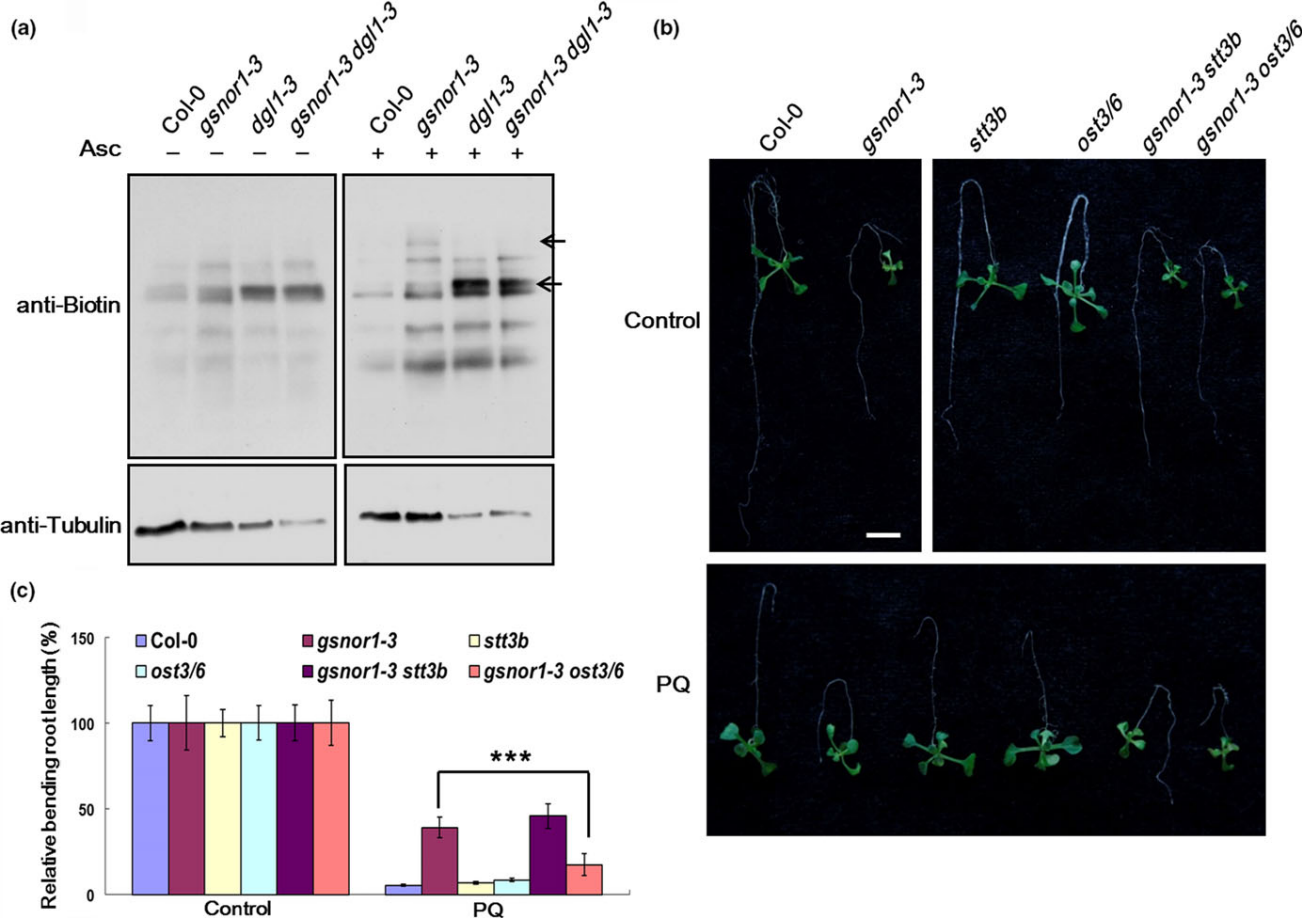
Biotin‐switch assay and paraquat resistance analysis. (a) In vivo biotin‐switch assay of 7‐day‐old seedlings. Arrowheads indicate different bands of proteins between *gsnor1‐3* and *gsnor1‐3 dgl1‐3*. (b) 7‐day‐old seedling germinated and vertically grown on 1/2 MS medium were transferred to 1/2 MS medium without (Control) or with 0.1 μM paraquat (PQ) in inverted orientation for additional 5 days. Bar = 5 mm. (c) Relative bending root lengths of seedlings in panel (b). Asterisks indicate significant differences (***p < 0.001, Student's *t* test)

Using biotin‐switch analysis, we checked the *S*‐nitrosylation patterns in relative mutants. In line with previous research, *gsnor1‐3* had much more bands representing nitrosylated proteins, but here we cannot tell the level difference of *S*‐nitrosylation between *gsnor1‐3* and *gsnor1‐3 dgl1‐3* as some bands in *gsnor1‐3 dgl1‐3* were missing while some bands were aggravated. However, the profiles of *S*‐nitrosylation were apparently different between *gsnor1‐3* and *gsnor1‐3 dgl1‐3* (Figure 4a).

We checked the paraquat sensitivity of *gsnor1‐3 ost* double mutants by measuring the relative bending root lengths of 7‐day‐old seedlings transferred to 1/2 MS mediums containing 0.1 μM paraquat. The *gsnor1‐3 ost3/6* double mutant showed a hypersensitivity to paraquat while *gsnor1‐3 stt3b* still had a strong resistance (Figure 4b,c). As mentioned above, *stt3a stt3b* double mutant was embryo lethal while *stt3b* had no obvious underglycosylation defective phenotype, we suspect that because *stt3b* is a weak underglycosylation mutant that is not able to lead a paraquat sensitive phenotype.

### 3.6 Identification of TGG2 as a co‐substrate of *S*‐nitrosylation and *N*‐glycosylation

**Figure 5:**
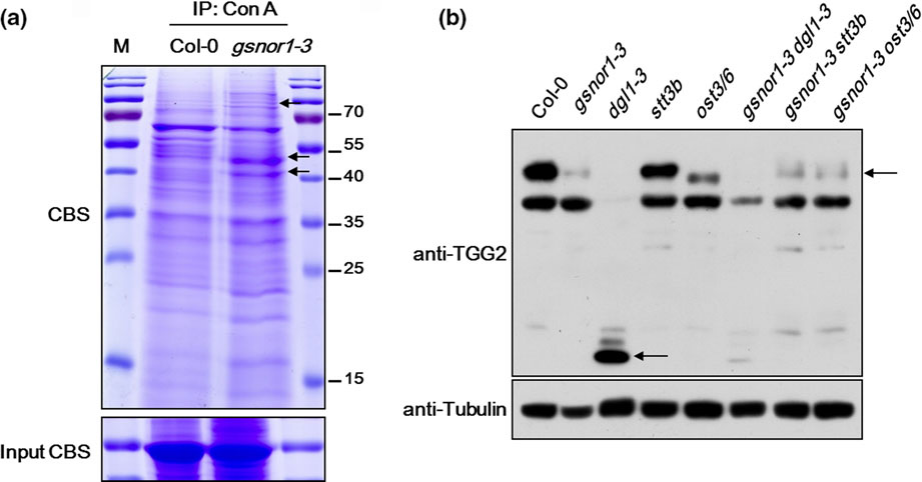
Affinodetection of specific glycoproteins in *gsnor1‐3* and *N*‐glycosylation analysis of TGG2. (a) Affinodetection with concanavalin A (Con A) of 7‐day‐old seedling proteins extracted from Col‐0 and *gsnor1‐3,* and separated on a 15% denaturating polyacrylamide gel. Arrowheads show altered glycoprotein profiles in *gsnor1‐3*. CBS for coomassie brilliant blue staining. (b) Immunoblotting of proteins extracts from 7‐day‐old seedlings with the antibody raised against TGG2. Arrowheads indicate TGG2 fragment in *dgl1‐3* and glycosylated TGG2 in *gsnor1‐3 ost3/6* compared to that in *ost3/6* and *gsnor1‐3 stt3b*

As described above, *dgl1‐3* suppressed the paraquat resistance and changed the *S*‐nitrosylation pattern of *gsnor1‐3* while *gsnor1‐3* rescued the embryo lethal phenotype of *dgl1‐3*; meanwhile the *S*‐nitrosylation and *N*‐glycosylation profiles were changed in *dgl1‐3* and *gsnor1‐3*. On the basis of these phenomena we suspected that *S*‐nitrosylation and *N*‐glycosylation crosstalks with each other through some co‐substrates. To identify the co‐substrates, the concanavalin A (Con A)‐conjugated agarose beads were used in immunoprecipitation (IP) to enrich distinct glycoproteins in *gsnor1‐3* compared to that in Col‐0 (Figure 5a, arrowheads). The proteins between 40 and 55 kDa were analyzed by mass spectrometry and cross referenced to the glycoprotein list of *Arabidopsis* reported in the *N*‐glycoproteomes research (Zielinska Dorota, Gnad, Schropp, Wiśniewski Jacek, & Mann, 2012). A total of 116 glycoproteins were identified and 22 proteins were also found in the list of *S*‐nitrosylated proteins reported in the *Arabidopsis* nitrosoproteomic analysis (Hu et al., 2015). Interestingly, nine out of 116 proteins related to unfolded protein reaction (UPR) such as CRT1, CRT3, PDILs were also identified (Table S1, Supporting Information). We picked TGG2 protein as a target for forward research because it had been proved to be a glycoprotein and the glycosylated and unglycosylated TGG2 proteins were easy to distinguish on a denaturating polyacrylamide gel by Western blot (Liebminger et al., 2012; Ueda et al., 2006). Using chemically synthesized antigen (sequence: AHALDPSPPEKLT) (Ueda et al., 2006), we prepared the antibody of TGG2.

As glycoproteins in underglycosylated mutants were fast degraded for misfolding through ERAD pathway, TGG2 protein volume in *ost3/6* was lower with higher electrophoretic mobility compared to that in Col‐0. However, no difference of TGG2 was observed between *stt3b* and Col‐0 suggesting that *stt3b* was a weak underglycosylation mutant which was coincident with the paraquat resistant phenotype (Figure 4b,c). TGG2 in *dgl1‐3* was suspected to be degraded to low molecular weight fragments (arrowheads) which were completely degraded in *gsnor1‐3 dgl1‐3*. In addition, TGG2 is a potential target of *S*‐nitrosylation which degrades faster in *gsnor1‐3*, it is possible that *S*‐nitrosylation promoted TGG2 degradation. As one *N*‐glycan side chain was removed from a glycoprotein, the protein molecular weight would minus 1 kDa, when comparing the electrophoretic mobility of TGG2 in *ost3/6, gsnor1‐3 stt3b,* and *gsnor1‐3 ost3/6*, TGG2 was obviously glycosylated in *gsnor1‐3 ost3/6* which had the same molecular weight as that in *gsnor1‐3 stt3b* intimating that *S*‐nitrosylation promotes *N*‐glycosylation of TGG2 and a crosstalk between *S*‐nitrosylation and *N*‐glycosylation involving co‐substrates.

## 4 DISCUSSION

In this study, by the isolation of paraquat sensitive mutant under *gsnor1‐3* background, we characterized a new mutation on *DGL1* nominated as *dgl1‐3*. Genetic and biochemical experiments showed that *dgl1‐3* suppressed the bushy phenotype and changed the *S*‐nitrosylation pattern of *gsnor1‐3*. As one important subunit of OST complex, it is believed that DGL1 functions in binding lipid‐linked oligosaccharide donor substrates (Pathak et al., 1995). The dysfunction of DGL1 in *Arabidopsis* (*dgl1‐1and 2*) causes severe development defects, such as embryo lethality, reduced cell elongation, and post‐embryonic development cease (Lerouxel et al., 2005), while rice *Osdgl1* mutant exhibited shorter root length, smaller root meristem, and cell death in the root (Qin et al., 2013). Here, we also observed that *dgl1‐3* was embryo lethal like *dgl1‐2* and ceased post‐embryonic development as *dgl1‐1*. Furthermore, the cell death level in the cotyledons of *dgl1‐3* was elevated. Both *dgl1‐1* and 2 are T‐DNA insertions in the promoter region while *Osdgl1* is a point mutation resulting in premature termination of protein synthesis. Using total cDNA of Col‐0 and *dgl1‐3* as PCR templates and primers for the full length of *DGL1* (1,314 bp), we amplified the same length of bands, and by qRT‐PCR the expression of *DGL1* in Col‐0 and *dgl1‐3* were at the same level (data not shown). Therefore, the development defects of *dgl1‐3* must be caused by dysfunction of DGL1 protein and the Gly186 is of great importance.

By segregation ratio assay of F2 population from different backgrounds, we further observed that *gsnor1‐3* rescued the embryo lethality phenotype of *dgl1‐3*. Furthermore, *gsnor1‐3 dgl1‐3* double mutant was reproductive meaning *gsnor1‐3* rescued the post‐embryo cease phenotype of *dgl1‐3*. DGL1 has two cysteine residues, by assuming that these cysteine residues were *S*‐nitrosylated and transforming cysteine to serine mutated DGL1 to *dgl1‐3* background, the transformed plant developed normally (data not shown). Therefore, it is impossible for *S*‐nitrosylation rescuing the dysfunction of DGL1, not to mention that DGL1 is just a subunit of OST complex and its *S*‐nitrosylation is hardly to influence the enzyme activity of OST during the *N*‐glycosylation reaction in *gsnor1‐3 dgl1‐3*.

Both *S*‐nitrosylation and *N*‐glycosylation are post‐translational modifications with enormous amount of substrates. As the *S*‐nitrosylation and *N*‐glycosylation patterns were changed in *dgl1‐3* and *gsnor1‐3*, the *gsnor1‐3 dgl1‐3* also showed the intermediate phenotypes, we hypothesized that *S*‐nitrosylation and *N*‐glycosylation might crosstalk with each other through some co‐substrates, but what is the possible molecular mechanism of the crosstalk? We speculated that disulfide bonds might play a critical role. *S*‐nitrosylation is commonly regarded as stable regulatory modification; however, Wolhuter et al. (2018) showed that *S*‐nitrosothiols rapidly react with thiols to form disulfides. That is, rat aortic smooth muscle cells treated with NO donor, transient *S*‐nitrosothiols were formed and rapidly transited to disulfides. Cherepanova, Shrimal, and Gilmore (2014) reported that in HeLa cells many *N*‐glycosylation sites were either closely bracketed by disulfide or that the *N*‐glycosylation sequon (NXT/S) contained a disulfide‐bonded cysteine as the X residue. In the *N*‐glycosylation process a transient mixed disulfide between the OST complex and a free thiol in a glycoprotein substrate was formed that delayed disulfide bond formation until the glycan was added to the Asparagine residue; disulfide in a nascent polypeptide can also be opened by OST to form a mixed disulfide allowing access of OST to an inaccessible sequon (Cherepanova et al., 2014). The work of Wolhuter and Cherepanova reminded us a question: what if the thiols near *N*‐glycosylation sequons of the co‐substrates were occupied by *S*‐nitrosothiols? Especially in *gsnor1‐3* which accumulated much more GSNO and *S*‐nitrosothiols in the cells, it is possible that the equilibrium between *S*‐nitrosothiols and disulfides could have been broken or both on an elevated level. We suspected that the occupancy of the thiols near *N*‐glycosylation sequons should consume less energy and facilitate the formation of mixed or bracketed disulfides than *N*‐glycosylation. The way to test and verify this hypothesis is to make *TGG2* transgenic plants with *S*‐nitrosylated sites mutations then to check in vivo if the mutated sites have infected on *N*‐glycosylation and vice versa. This could be our next work to deal with but the exact *S*‐nitrosylation and *N*‐glycosylation sites of TGG2 should be identified first.

Analysis of consensus sequence of *S*‐nitrosylated peptides revealed that the nitrosylated cysteine residues are usually flanked in motifs that contain acidic, but not basic, amino acid residues (Hu et al., 2015). In addition, the cysteine thiol micro‐environment has been proposed important factors for the specificity of protein *S*‐nitrosylation. Hao, Derakhshan, Shi, Campagne, and Gross (2006) suggested that the key determinants of *S*‐nitrosylation specificity are likely to be undefined 3‐D structural features. As *N*‐glycosylation plays an important role in glycoprotein quality‐control (GQC) system and controls the primary structure of nascent polypeptides, we have reasons to believe that underglycosylation might lead to a change in the 3‐D structures of peptides and effects on *S*‐nitrosylation. On the other hand, the destination of underglycosylated proteins are finally degraded, thus *S*‐nitrosylation of these proteins should be a waste of energy, unless in the cells of *gsnor1‐3* where accumulated too much GSNO.

Interestingly, we can also conclude from the reports mentioned above that in time sequence *N*‐glycosylation and *S*‐nitrosylation should occur before the formation of disulfide bonds, but what is the sequence between *N*‐glycosylation and *S*‐nitrosylation? It is known that *N*‐glycosylation is actually a co‐translational modification: when a nascent glycopeptide is translated by membrane‐bound ribosomes and enters the ER lumen, it is glycosylated by ER membrane anchored OST complex. Dose *S*‐nitrosylation happen even before *N*‐glycosylation? It is mysterious to answer this question.

In our previous work, we assumed that *PAR2/GSNOR1/HOT5* functions downstream of superoxide to regulate cell death (Chen et al., 2009). Here, because H_2_O_2_ as a superoxide in *dgl1‐3* was depressed in *gsnor1‐3 dgl1‐3,* it seems our assumption was reasonable.

## 5 CONCLUSION

By characterizing the suppressor of *gsnor1‐3*, we identified a new point mutation on the *DGL1* gene which genetically repressed the paraquat resistance of *gsnor1‐3*. We also presented genetic and biochemical evidences to shed light on the crosstalk between *S*‐nitrosylation and *N*‐glycosylation based on co‐substrates. As TGG2 was one of the co‐substrates of *S*‐nitrosylation and *N*‐glycosylation, the elevated *S*‐nitrosylation level rescued its underglycosylation in *gsnor1‐3 ost3/6* double mutant. We hypothesized that the transformation between *S*‐nitrosothiols and disulfides bracketing *N*‐glycosylation sites regulated the crosstalk between *S*‐nitrosylation and *N*‐glycosylation, but further investigation are needed.

## AUTHOR CONTRIBUTIONS

J.Z. designed the study; H.D. performed the experiments, data analysis, and wrote the article; L.C. constructed the EMS‐mutant library; N.Z., J.M., and B.R. provided necessary assistance in the study.
