# Supplementary material for: A new insight to explore the regulation between S‐nitrosylation and N‐glycosylation
Source: Plant Direct. 2019 Mar 1;3(2):e00110. doi: 10.1002/pld3.110 (PMC6508853; doi:10.1002/pld3.110)
Supplement: Supplementary file 1 [file PLD3-3-e00110-s001.pdf]

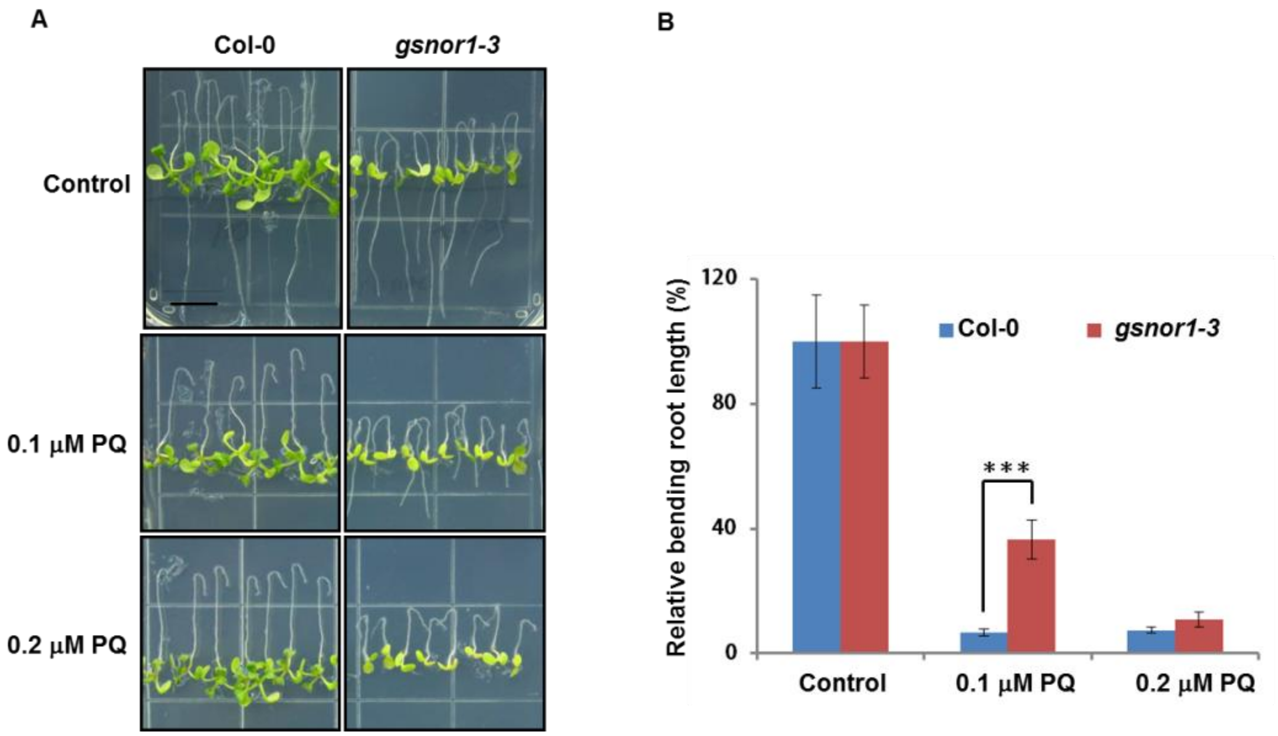

Supplemental Figure S1 The proper concentration of paraquat used for screening the suppressor of *gsnor1-3*

A: 7-day-old Col-0 and *gsnor1-3* seedlings grown on 1/2 MS medium were transfer to 1/2 MS mediums without (Control) or with variant concentration of PQ for additional 5 days. On medium containing 0.1 μM PQ *gsnor1-3* showed resistance to paraquat. Bar = 1.0 cm.

B: The relative bending root length of seedlings in panel A. Asterisks indicate significant differences (***P < 0.001, Student's t test).
