# Supplementary material for: A new insight to explore the regulation between S‐nitrosylation and N‐glycosylation
Source: Plant Direct. 2019 Mar 1;3(2):e00110. doi: 10.1002/pld3.110 (PMC6508853; doi:10.1002/pld3.110)
Supplement: Supplementary file 2 [file PLD3-3-e00110-s002.pdf]

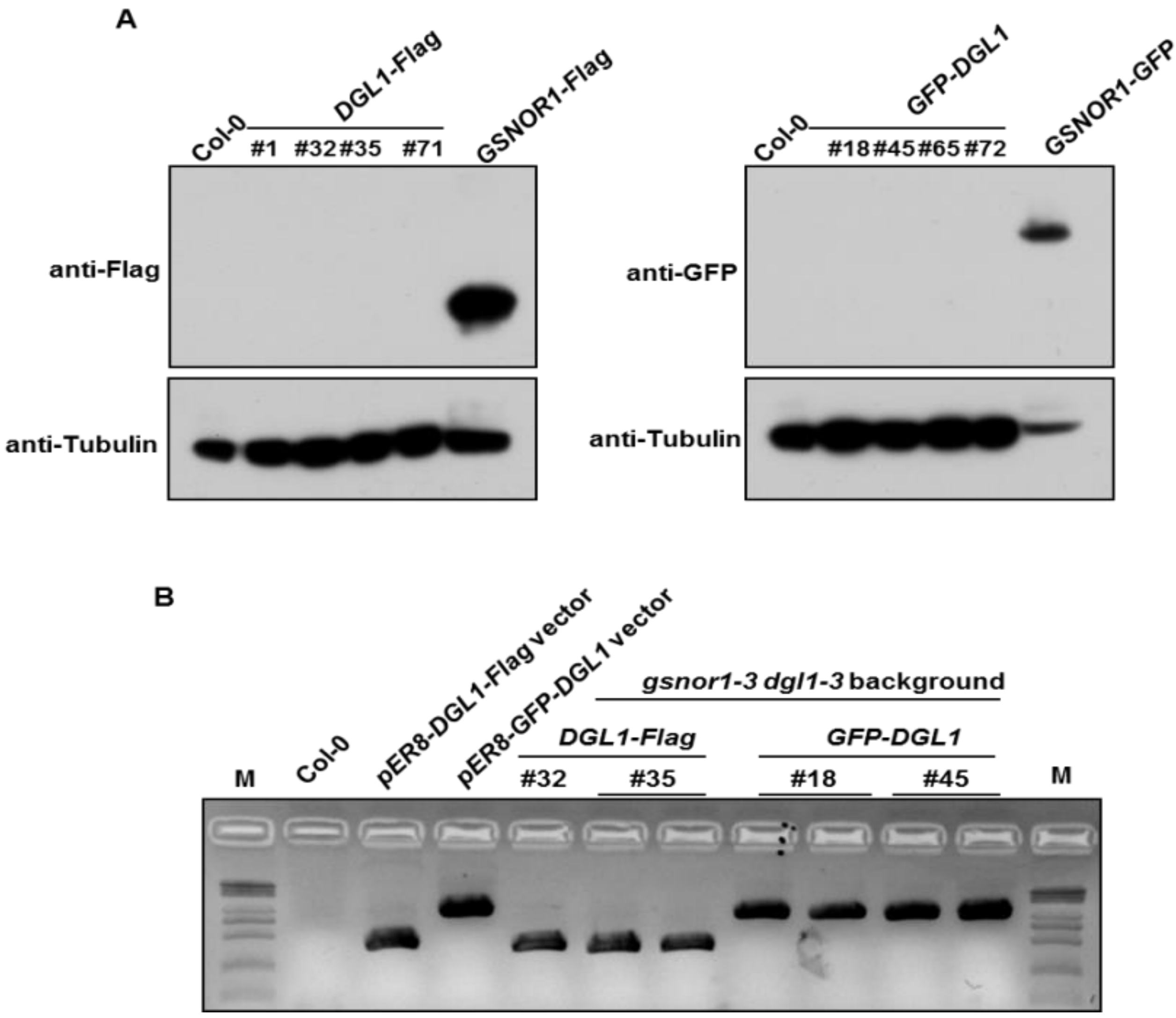

Supplemental Figure S2 Western blot and PCR analyses of integration of *DGL1*

A: Proteins extracted from Col-0 and Flag or GFP tagged DGL1, GSNOR1 transgenic plants were analyzed by Flag and GFP antibodies. Col-0 and GSNOR1 transgenic plants were set as negative and positive controls.

B: Electrophoresis profiles of PCR products amplified by primers anchored on the tags (Flag/GFP) and *DGL1*. The genomic DNA extracted from Col-0 and transgenic plants were used as modules. The Col-0 and vectors used to transform *gsnor1-3 dgl1-3* plants were used as negative and positive controls.
