# Supplementary material for: A new insight to explore the regulation between S‐nitrosylation and N‐glycosylation
Source: Plant Direct. 2019 Mar 1;3(2):e00110. doi: 10.1002/pld3.110 (PMC6508853; doi:10.1002/pld3.110)
Supplement: Supplementary file 4 [file PLD3-3-e00110-s004.pdf]

Dear Editor:

Thank you for reviewing my manuscript "A new insight to explore the regulation between *S*-nitrosylation and *N*-glycosylation", the following are my responses to the comments in detail:

Comment 1) Carefully check the entire manuscript for the English language.

Response: According to the comments of reviewer #1 and #3, the typos and mistakes were corrected. And the manuscript has been sent and revised by a native English-speaking person. A new Figure 1 PDF document was also prepared with adjusted column description on X-axis of part G.

Comment 2) R**evise the Discussion section as commented by the reviewer one of Plant Physiology** (Reviewer #1: As a major points, the discussion must be substantially improved in extent and detail, in the actual state author rather shortly discuss results published in previous papers of other groups. Moreover, when authors cite the recent paper of Wolhuter et al., which suggest that S-nitrosylation has no relevance as signaling mechanism in vivo, how than they would interpret their finding with gsnor mutant lines?).

Response: We have added the discussion in detail of the functional importance of DGL1 protein and the reasons why we thought the study of co-substrates might be a breakthrough to decipher the relationship between *S*-nitrosylation and *N*-glycosylations.

In the work of Wolhuter et al., there is no suggestion that *S*-nitrosylation has no relevance as signaling mechanism, actually, *S*-nitrosylation is a commonly known protein modification which manipulates the functions of proteins. Wolhuter et al. mainly suggested that formation of *S*-nitrosation is not an end of signaling but the disulfides formed by rapid reaction of *S*-nitrosothiols and thiols are. On the other hand, Wolhuter's work was carried out in animal models with existence of Nitric Oxide Synthases (NOS) which had never been found in plants. Thus if anyone wants to check our hypothesis in animal systems, we think they just need to add NO donor into cell cultures or use cell lines with elevated NOS activity then analyze the *N*-glycosylation patterns of these cells with specialized antibodies.

Comment 3) **Consider the suggestion made by reviewer two of Plant Physiology on proteomic data deposition** (Reviewer #2: The proteomic data are not adequately reported. Raw data require deposition in a public repository, information pertaining to peptide numbers, scores, instrumentation, data analysis, etc etc etc etc have not been included.).

Response: We have prepared a new excel file about the peptides identified by MS with gene IDs, peptide IDs, protein descriptions and sequence coverage. The methods we carried out the mass spectrometry and proteomic row data analysis and Biotin-switch and western-blot assays were added into the Method section
